# Single-Stage Extraction and Separation of Co^2+^ from Ni^2+^ Using Ionic Liquid of [C_4_H_9_NH_3_][Cyanex 272]

**DOI:** 10.3390/molecules27154806

**Published:** 2022-07-27

**Authors:** Xiaohua Jing, Zhumei Sun, Dandan Zhao, Huimin Sun, Jie Ren

**Affiliations:** 1 School of Chemistry and Chemical Engineering, Anyang Normal University, Anyang 455000, China; zhaodandan202009@126.com; 2 Henan Province Key Laboratory of New Optoelectronic Functional Materials, Anyang 455000, China; 3 International Joint Laboratory of Henan Photoelectric Functional Materials, Anyang 455000, China; 4 School of Environment and Safety Engineering, North University of China, Taiyuan 030051, China; sunzhumei41@nuc.edu.cn (Z.S.); 01726@aynu.edu.cn (H.S.); jsrencn@gmail.com (J.R.)

**Keywords:** extraction, ionic liquid, separation factor, Co^2+^ from Ni^2+^, response surface methodology

## Abstract

The purpose of this study was to optimize the extraction conditions for separating Co^2+^ from Ni^2+^ using N-butylamine phosphinate ionic liquid of [C_4_H_9_NH_3_][Cyanex 272]. A Box–Behnken design of response surface methodology was used to analyze the effects of the initial pH, extraction time, and extraction temperature on the separation factor of Co^2+^ from sulfuric acid solution containing Ni^2+^. The concentrations of Co^2+^ and Ni^2+^ in an aqueous solution were determined using inductively coupled plasma-optical emission spectrometry. The optimized extraction conditions were as follows: an initial pH of 3.7, an extraction time of 55.8 min, and an extraction temperature of 330.4 K. The separation factor of Co^2+^ from Ni^2+^ under optimized extraction conditions was 66.1, which was very close to the predicted value of 67.2, and the error was 1.7%. The equation for single-stage extraction with high reliability can be used for optimizing the multi-stage extraction process of Co^2+^ from Ni^2+^. The stoichiometry of chemical reaction for ion-exchange extraction was also investigated using the slope method.

## 1. Introduction

Cobalt is a strategic metal that has many industrial applications, e.g., rechargeable batteries, superalloys, cemented carbide, and colorants [1,2]. This metal can be extracted or recycled from natural and secondary resources, e.g., Ni-Co laterites [3], stainless steel scarps [4], and spent lithium–ion batteries [5]. Therefore, to obtain the cobalt products, a leaching process is needed [6]. The multicomponent mixtures of metal ions, e.g., Co^2+^, Ni^2+^, Mn^2+^, Al^3+^, Zn^2+^, and others, are coexisting in the leaching solutions [4]. Efficient separation of Co^2+^ from leaching solution containing Ni^2+^ is the key technology for the final preparation of high-purity cobalt or nickel products [7,8].

As a matter of fact, two metals, cobalt and nickel, are difficult to completely separate due to similar physicochemical properties [9,10]. Based on a novel scale micro-reactor, the efficient separation of Co^2+^ from Ni^2+^ in sulfuric acid solution was achieved using the organic extractant of Cyanex 272, and this separation method was a two-stage counter-current extracted process [11]. Using the five-stage extracted process, the separation factor of Co^2+^ from Ni^2+^ (*β*_Co/Ni_) could be reached 9.5 × 10^3^ using the new organic extractant of di-decylphosphinic acid [12]. About >99% of Co^2+^ and 11% of Ni^2+^ were extracted using the extractant of primene JMT-Cyanex 272 with the process of four-stage counter extraction [13], and the efficient separation of the two metal ions was achieved based on this process. In addition, extractants of MEXTRAL 507P [14], INET-3 [14], and PC88A [15] were also used for the separation of Co^2+^ from Ni^2+^. According to the above published articles, the efficient separation of Co^2+^ from Ni^2+^ in sulfate solution was generally realized using multi-stage extraction, and the experimental conditions of single-stage extraction were one of the essential factors for designing multi-stage extraction.

However, solvent extraction using organic extractants has several drawbacks, such as the irreversible loss of the organic phase [16] and easy volatilization of extractants [17]. For example, the oxidation reaction of primary amine N1923 may occur in the separation of V(V) from the leaching solution, and this process results in irreversible loss of organic extractant [18]. In addition, some organic extractants are easily volatile during extraction, and the organic vapors that fill the workshop may harm workers and cause environmental pollution problems [19]. Therefore, the demerits of solvent extraction using common organic extractants may lead to increased production costs and environmental problems [20,21]. How to reduce or avoid these problems in scientific research is one of the current research directions [22]. 

To our best knowledge, ionic liquids (ILs) have been used in solvent extraction for separating metal ions [4,23,24] due to their unique properties, i.e., low vapor pressure, high dissolving capability, chemical/thermal stability [25], and designable structure [26,27]. For example, the efficient and sustainable separation of Ho, Er, and Lu from Y was achieved using the functionalized ionic liquid of [N_1888_][NDA] [28]; the new ionic liquid of [N_14111_][DBP] exhibited high efficiency in uranium separation [29]; and the extraction efficiencies of Cd(II), Cu(II), and Zn(II) were about 85, 67, and 69% using the hydrophobic trioctylammonium-based ionic liquid of [HTOA][adipate] [30]. If more ionic liquids are used as extracted agents for the separation of Co^2+^ from Ni^2+^, the environmentally friendly separation process may be further investigated based on the single-stage extraction [31], and different kinds of ionic liquids may be applied in the recovery of metal ions from secondary waste resources.

With the aim of reducing the irreversible loss of the organic phase and decreasing the environmental pollution in the extraction process, the task-specific ionic liquid (TSIL) of [C_4_H_9_NH_3_][Cyanex 272] was synthesized using a one-step reaction. The FT-IR, ^1^H NMR, and ^13^C NMR of TSIL were made with the aim of analysis of purity and impurities. The optimization of extraction conditions for the separation of the two metal ions was determined by the Box–Behnken design (BBD) [32], which is a typical method of response surface methodology (RSM) [32]. Optimization of the extraction conditions for metal ions, e.g., Cu^2+^ [33], Cr^3+^ [34], and V^4+^ [35], were successfully performed using the BBD. The BBD was used to optimize the initial pH value (pH_ini_), extraction time (*t*_e_), and extraction temperature (*T*_e_) with the aim of achieving the maximum separation factor of Co^2+^ from Ni^2+^ (*β*_Co/Ni_). In addition, the extraction mechanism was also investigated using the slope method according to the published papers.

## 2. Materials and Methods

### 2.1. Chemicals and Reagents

**Figure 1:**
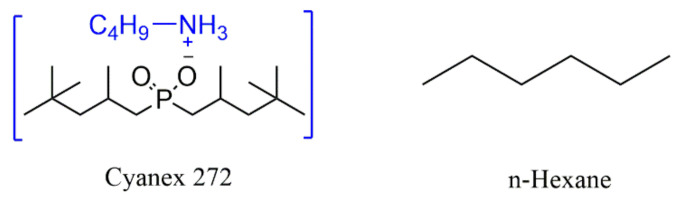
Structure of extractant and diluent.

The bis(2,4,4-trimethylpentyl) phosphinic acid (Cyanex 272, C_16_H_35_O_2_P, purity: ≥92 wt%) was purchased from Zhengzhou Hecheng New Materials Tech Co., Ltd. (Zhengzhou, China). Butylamine (C_4_H_9_NH_2_, purity: ≥99 wt%), Sodium hydroxide (NaOH, purity: ≥92 wt%), n-Hexane (C_6_H_14_, purity: ≥99 wt%), Anhydrous ethanol (≥99 wt%), NiSO_4_·6H_2_O (purity: ≥98.5 wt%,) CoSO_4_·7H_2_O (purity: ≥98 wt%), and H_2_SO_4_(purity: ≥98 wt%) were purchased from Shanghai Titan Scientific Co., Ltd. (Shanghai, China). Standard solutions of cobalt and nickel (1000 μg/mL) were purchased from Shanghai Macklin Biochemical Technology Co., Ltd. (Shanghai, China). The synthesized [C_4_H_9_NH_3_][Cyanex 272] and n-Hexane were used as extractant and diluent (Figure 1), respectively. All the chemicals and reagents were of reagent grade and used without further purification unless otherwise stated.

The simulated sulfuric acid solution containing 14 g/L of Co^2+^ and 14g/L of Ni^2+^ [4,23] was prepared by dissolving the NiSO_4_·6H_2_O and CoSO_4_·7H_2_O in deionized water. With the aim of obtaining a purity solution, the simulated solutions should pass through a filtration membrane with an aperture of 0.45 microns. The pH_ini_ of the simulated solutions was adjusted by adding H_2_SO_4_ or NaOH solutions. 

### 2.2. Analytical Methods

The spectra of FT-IR were obtained using a SEPCTRUM GX1 instrument (PerkinElmer, Waltham, MA, USA) at room temperature. The ^1^H and ^13^C NMR was obtained using an NMR spectrometer (Avance-III 400 MHz, Bruker, Zurich, Switzerland). A pH meter (Delta 320, Zurich, Switzerland) with an uncertainty of 0.01 was used to measure the pH_ini_ of the solutions. The concentrations of the two metal ions were analyzed using inductively coupled plasma-optical emission spectrometry (ICP-OES, Optima 8000, PerkinElmer, Waltham, MA, USA). The simulated solutions containing Co^2+^ and Ni^2+^ were diluted by deionized water to the concentrations of 0.1–40 ppm for the next tests.

### 2.3. Synthesis of the TSIL

**Figure 2:**
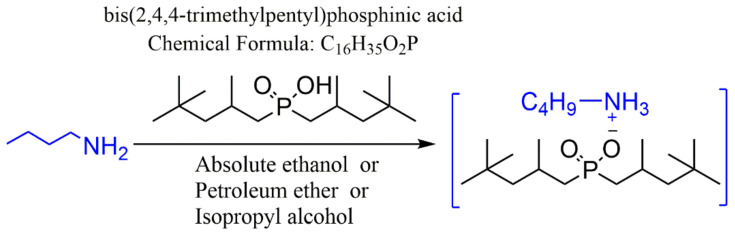
Synthetic reaction of [C_4_H_9_NH_3_][Cyanex 272].

The [C_4_H_9_NH_3_][Cyanex 272] was synthesized in one step reaction (Figure 2), and the reaction process was observed by thin layer chromatography (TLC silica gel 60 F-254 on aluminum plate, Merck, NJ, USA). The spectra of the product are provided in the Supplementary Materials.

### 2.4. Single-Factor Experiment

The experiments were carried out by mixing an equal volume of the organic phase and simulated solution (each 15 mL) in a separating funnel using a water-bath thermostatic oscillator (SHA-C, Jie Rui Er, Changzhou, China). When the extraction was completed, the two immiscible phases were separated for analysis. The pH values in the sulfuric acid solution before and after extraction were determined using a pH meter. The concentrations of two metals before and after extraction were determined by ICP-OES. The effects of pH_ini_, *t*_e_, and *T*_e_ were first studied using single-factor experiments as follows: one extraction condition was changed when the other conditions were kept constant in every experiment, and the influences of each condition were studied by analyzing the concentration of metal ions in the solutions. All experiments were carried out three times, and the average values were obtained for the diagraph analysis under normal pressure unless otherwise stated. 

### 2.5. Optimizing the Extraction Conditions by BBD

Design-Expert Ver. 8.0.6 (Trial version, Stat-Ease, Minneapolis, MN, USA) was used to optimize the extraction conditions [36]. According to the single-factor experimental results, three independent factors, i.e., pH_ini_ (A), *t*_e_ (B), and *T*_e_ (C), were found to be responsible for the *β*_Co/Ni_. With the aim of analyzing the A, B, and C, three-factor and three-level Box–Behnken experimental design was used to conduct response surface analysis [37], and the three factors were optimized at three levels (−1, 0, −1) (Table 1). 

Based on the experimental data, regression analysis was made using the following second-order polynomial equation:

(1) $$\begin{array}{l} \beta_{\mathrm{Co}/\mathrm{Ni}}=D_{0}+D_{A}A+D_{B}B+D_{C}C\;+\\ \;D_{A}{}_{A}A^{2}+D_{B}{}_{B}B^{2}+D_{C}{}_{C}C^{2}+\\ \;D_{A}{}_{B}AB+D_{B}{}_{C}BC+D_{A}{}_{C}AC \end{array}$$

where *D*_0_ is a constant coefficient; *D*_A_, *D*_B_*,* and *D*_C_ represent coefficients of the linear equation for three independent factors, i.e., pH_ini_ (*A*), *t*_e_ (*B*), and *T*_e_ (*C*). *D*_AA_, *D*_BB_*,* and *D*_CC_ represent coefficients of the quadratic equation for three factors, i.e., *A*^2^, *B*^2^, and *C*^2^. *D*_AB_, *D*_BC_*,* and *D*_AC_ represent coefficients of interaction effects of variables, i.e., *AB*, *BC*, and *AC.*

The significance of the equations was studied according to analysis of variance (ANOVA). The significance of each coefficient and the interaction between the independent variables were also investigated using the obtained *p*-value. In addition, real experiments were made to evaluate the accuracy of the optimized extraction conditions.

### 2.6. Calculations

The extraction percentages of Co^2+^ (*E*_Co_), Ni^2+^ (*E*_Ni_), and the *β*_Co/Ni_ were calculated as Equations (2)–(4).

(2) $$E_{\mathrm{Co}}=\frac{V_{1}C_{1\left(\mathrm{Co}\right)}-V_{2}C_{2(\mathrm{Co})}}{V_{1}C_{1\left(\mathrm{Co}\right)}}\times 100$$

(3) $$E_{\mathrm{Ni}}=\frac{V_{1}C_{1\left(Ni\right)}-V_{2}C_{2(\mathrm{Ni})}}{V_{1}C_{1\left(\mathrm{Ni}\right)}}\times 100$$

(4) $$\beta_{\mathrm{Co}/\mathrm{Ni}}=\frac{E_{\mathrm{Co}}\times (100-E_{\mathrm{Ni}})}{(100-E_{\mathrm{Co}})\times E_{\mathrm{Ni}}}$$

where *V*_1_ (L) and *V*_2_(L) represent the volumes of the stock and raffinate solution, respectively. *C*_1(Co)_ (g/L), *C*_2(Co)_ (g/L), *C*_1(Ni)_ (g/L), and *C*_2(Ni)_ (g/L) represent the concentrations of Co^2+^ and Ni^2+^ in the stock and raffinate solution.

## 3. Results and Discussion

### 3.1. ^1^H, ^13^C NMR, and FT-IR of [C_4_H_9_NH_3_][Cyanex 272]

**Figure 3:**
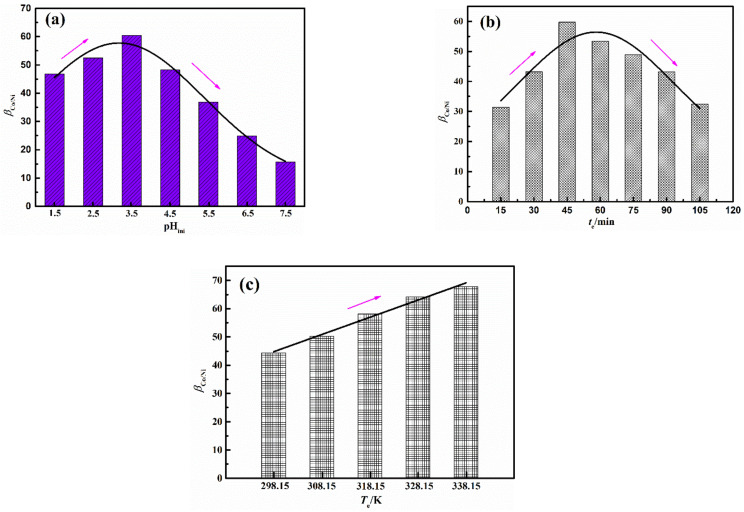
Effect of pH_ini_ on *β*_Co/Ni_ (**a**); effect of *t*_e_ on *β*_Co/Ni_ (**b**); effect of *T*_e_ on *β*_Co/Ni_ (**c**).

The bis(2,4,4-trimethylpentyl) phosphinic acid, i.e., Cyanex 272 (2.93 g, 10 mmol) and butylamine (0.74 g, 10 mmol) in absolute ethanol were stirred for 3.5 h at 88 ℃ under the reflux condition. A light-yellow oily liquid was obtained with a 93.5% yield after evaporation of solvent using the rotary evaporation system. ^1^H NMR (400 MHz, CDCl_3_, ppm, C_4_H_9_NH_2,_ Supplementary Materials Figure S1): 0.92(t, 3H, 1 × CH_3_); 1.13(s, 2H, 1 × CH_2_); 1.35(m, 2H, 1 × NH_2_); 1.43(m. 2H, 1 × CH_2_); and 2.69(t, 3H, 1 × CH_2_). ^1^H NMR ([C_4_H_9_NH_3_][Cyanex 272], Supplementary Materials Figure S2): 0.93(t, 21H, 7 × CH_3_), 1.10 (t, 9H, 2 × CH_3_ + 1 × NH_3_), 1.36(m, 8H, 4 × CH_2_), 1.57(d, 2H, 2 × CH), 1.69(m, 2H, 1 × CH_2_), 1.89(s, 2H, 1 × CH_2_), 2.78(t, 2H, 1 × CH_2_). In the synthetic feedstock of n-butylamine (C_4_H_9_NH_2_), the shift of hydrogen on -NH_2_ is about 1.35 ppm, with a small widening on the right side of the peak. For the synthesized ionic liquid of [C_4_H_9_NH_3_][Cyanex 272], compared with the C_4_H_9_NH_2_, the -NH_2_ becomes -NH_3_, and the probability of hydrogen shift on the -NH_3_ is within the range of 1.11pm (Figure 3b). The changing shift of hydrogen on the -NH_3_ for synthesized ionic liquid resulted from the formation of new bonds in the ionic liquid. ^13^C NMR (400 MHz, CDCl_3_, ppm, Supplementary Materials Figure S3): 13.06(s, 1C, 1 × CH_3_), 20.07(s, 1C, 1 × CH_2_), 24.04(m, 2C, 2 × CH_3_), 25.50(d, 2C), 30.24(t, 7C, 6 × CH_3_ + 1 × CH_2_), 30.38(d, 2C, 2 × CH), 39.37(s, 1C, 1 × CH_2_), 40.88(d, 1C, 1 × CH_2_), 41.76(d, 1C, 1 × CH_2_), 53.53(m, 2C, 2 × CH_2_). FT-IR (cm^−1^, Supplementary Materials Figure S4): asymmetric stretching vibration peak of methyl group (2950.60 cm^−1^), asymmetric stretching vibration peak of ethyl group (2900.46 cm^−1^), symmetric stretching vibration peak of methyl group (2868.16 cm^−1^), in-plane vibration peak of amino group (1635.51 cm^−1^), Symmetric deformation peak of ethyl group (1466.30 cm^−1^), Symmetric deformation peak of ethyl group of methyl group (1363.45 cm^−1^), stretching vibration peak of carbon–carbon bond (1126.73, 1022.10 cm^−1^).

### 3.2. Single-Factor Experiment 

The concentration of [C_4_H_9_NH_3_][Cyanex 272] was set as 0.24 mol/L considering the concentration of Co^2+^ in the solution is 0.24 mol/L [4]. With the volume ratio of organic phase to the aqueous phase (O/A) = 1:1, the effect of pH_ini_ on *β*_Co/Ni_ is shown in Figure 3a when *t*_e_ = 60 min and *T*_e_ = 303.15 K. The *β*_Co/Ni_ first increases and then decreases with the growth of pH_ini_. According to Equation (4), the *β*_Co/Ni_ is proportional to *E*_Co_ and inversely proportional *E*_Ni_, and the final value of *β*_Co/Ni_ is determined by the ratio of *E*_Co_ and *E*_Ni_.

The influence of *t*_e_ on *β*_Co/Ni_ is shown in Figure 3b when *T*_e_ = 303.15 K, pH_ini_ = 3.5, and O/A = 1:1. The *β*_Co/Ni_ first increases and then decreases with the increase in *t*_e_ due to the different extraction kinetics of Co^2+^ and Ni^2+^ [38]. The effect of *T*_e_ on *β*_Co/Ni_ is shown in Figure 3c when *t*_e_ = 45 min, pH_ini_ = 3.5, and O/A = 1:1. The *β*_Co/Ni_ increases with the growth of *T*_e_. The reason is that higher temperature reduces the viscosity and surface tension of the extraction system, which is beneficial to mass transfer [37]. In addition to this, the extraction reaction of Co^2+^ using extractant of [C_4_H_9_NH_3_][Cyanex 272] is an endothermic reaction, and the higher *T*_e_ is conductive to the reaction [10]. However, considering the cost of the extraction process, the highest temperature in the next experimental design was set at 333.15 K. 

### 3.3. Optimization of the Conditions for Extraction Process

Seventeen experiments were first designed using BBD and then carried out one by one, and the results of these experiments are listed in Table 2**,** which was used to obtain the predicting equation. At least one variable is different in each group of experiments, and three levels of pH_ini_ are 1.50, 3.75, and 6.00, and three levels of *t*_e_ are 15.00, 52.50, and 90.00 min, and three levels of *T*_e_ are 298.15, 315.65, and 333.15 K, and the biggest value of *β*_Co/Ni_ is 65.51 when pH_ini_ = 3.75, *t*_e_ = 52.50, and *T*_e_ = 315.65. According to a regression analysis from the experimental data, the *β*_Co/Ni_ could be explained as Equation (5) according to second-order polynomial Equation (1). The *D*_0_ is 64.36; *D*_A_, *D*_B_*,* and *D*_C_ are 1.39, 5.68, and 4.91; *D*_AA_, *D*_BB_*,* and *D*_CC_ are −11.59, −22.07, and −3.54; *D*_AB_, *D*_BC_*,* and *D*_AC_ are −0.13, 0.44 and −0.20, respectively.

(5) $$\begin{array}{l} \beta_{\mathrm{Co}/\mathrm{Ni}}=64.36+1.39A+5.68B+4.91C\\ \;-0.13AB-0.20AC+0.44BC\\ \;-11.59A^{2}-22.07B^{2}-3.54C^{2} \end{array}$$

The variance of the quadratic prediction Equation (5) is summarized and listed in Table 3. The determination coefficient (*R*^2^ = 0.99) of Equation (5) is close to 1, which indicates that the effective correlation between predicated and measured values are obtained using this equation. The adjusted determination coefficients (adj *R*^2^ = 0.98) are also close to 1, which demonstrates that the equation is significant for accurately predicting the *β*_Co/Ni_. In addition to this, the *F*-value is 159.51 and the *p*-value < 0.0001, which implies that the equation is significant too. The *p*-value of the lack of fit is 0.0548, which indicates that the lack of fit is insignificant compared to the pure error. The lower coefficient of variation (C.V.% = 3.25) indicated that small deviation and high reliability of the experimental value. The regression equation can be used for predicting the experiment results instead of the real extractive experiments, and it can also be used to predict and analyze the *β*_Co/Ni_. 

**Figure 4:**
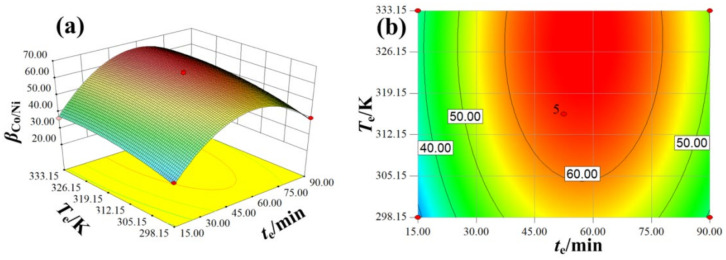
The 3D response surface and projection of *β*_Co/Ni_ affected by *t*_e_ (**a**) and *T*_e_ (**b**).

The values of *A*, *B*, *C*, *A*^2^, *B*^2^, and *C*^2^ are significant (*p* < 0.05), and the other values are not significant (*p* > 0.05). The influence degree of various factors on the *β*_Co/Ni_ is ranked in the following order: *C* > *B* > *A*. The coefficients of two factors, i.e., *AB*, *AC*, and *BC*, are 0.8668, 0.8028, and 0.5835, respectively. The effect degree of interaction based on two factors on the *β*_Co/Ni_ is ranked as *BC* > *AC > AB.* The *BC* has the biggest effect on the *β*_Co/Ni_ compared with the other two interaction factors of *AC* and *AB*, and the influent of *BC* on *β*_Co/Ni_ is shown in Figure 4. As shown in Figure 4b, the red zone represents the higher *β*_Co/Ni_ under the different *T*_e_ and *t*_e_, and the efficient separation of the two metal ions will be operated according to the equation prediction.

### 3.4. Verification of Equation

**Figure 5:**
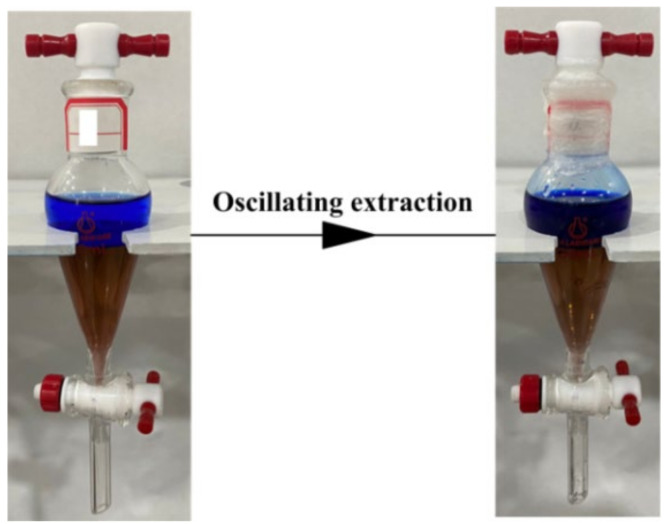
Single-stage extraction for verification experiment.

Based on the quadratic equation, the optimum extraction conditions of Co^2+^ from Ni^2+^ were calculated as follows: a pH_ini_ of 3.7, a *t*_e_ of 55.8 min, a *T*_e_ of 330.4 K, and the predicted *β*_Co/Ni_ of 67.2. The verification experiments (Figure 5) were tested with the optimum extraction conditions, and the actual *β*_Co/Ni_ was 66.1. The experimental value was close to the predicted value, and the experimental error was small. The experimental results indicate that Equation (5) can better predict the *β*_Co/Ni_ for single-stage extraction of Co^2+^ from Ni^2+^.

With the optimized single-stage extraction conditions, the separation factors of Co^2+^ from Ni^2+^ using Cyphos IL 104 [4], Cyanex 301 [39], Primene JMT-Cyanex 272 [13], and [C_4_H_9_NH_3_][Cyanex 272] are 14, 1.8, 45.3, and 67.2, respectively (Table 4). It is concluded that the [C_4_H_9_NH_3_][Cyanex 272] has better ability to efficient separate Co^2+^ from Ni^2+^ than Cyphos IL 104 [4], Cyanex 301 [39], and Primene JMT-Cyanex 272 [13]. In other words, the [C_4_H_9_NH_3_][Cyanex 272] is more effective in separating the two metal ions than three extractants according to their separation factors of Co^2+^ from Ni^2+^. The new TSIL of [C_4_H_9_NH_3_][Cyanex 272] is a kind of efficient extractant for the separation of the two metal ions.

### 3.5. Extraction Mechanism of [C_4_H_9_NH_3_][Cyanex 272]

The slope method can be used to determine the stoichiometry of chemical reactions for metal ion extraction [40,41]. The extraction equilibrium constant (*K*) and distribution ratio of the two phases (*D*) can be obtained using balanced chemical reactions. The [C_4_H_9_NH_3_][Cyanex 272] is a protic ionic liquid [42], and the cationic group of C_4_H_9_NH_3_]^+^ is very soluble in the aqueous phase (Supplementary Materials Figure S5), but the solubility of [C_4_H_9_NH_3_][Cyanex 272] in the aqueous phase is very poor (Supplementary Materials Figure S6, and the solubility is about 0.45 ± 0.02 g/100 g H_2_O at the temperature of 298.2 K and normal pressure). It is assumed that protons can be completely transferred during the extraction reaction. The extractive mechanism of Co^2+^ in the sulfuric acid medium by [C_4_H_9_NH_3_][Cyanex 272] is the ion-exchange reaction [43], and the chemical reaction can be expressed as Equation (6).

(6) $$m[C_{4}H_{9}{\mathrm{NH}}_{3}{][\mathrm{Cyanex}\;272]}_{\left(\mathrm{org}\right)}+{\mathrm{Co}}_{\left(\mathrm{aq}\right)}^{2+}\overset{K_{\mathrm{eq}}}{⇄}{\mathrm{Co}[\mathrm{Cyanex}\;272]}_{2}{}_{\left(\mathrm{org}\right)}+m{\left[C_{4}H_{9}{\mathrm{NH}}_{3}\right]}_{\left(\mathrm{aq}\right)}^{+}$$

The extractive equilibrium constant of Co^2+^ (*K*_Co_) can be listed as Equation (7) according to Equation (6).

(7) $$K_{\mathrm{Co}}=\frac{C_{1}\cdot {\left(C_{3}\right)}^{m}}{{\left(C_{2}\right)}^{m}\cdot C_{4}}$$

where the *C*_1_ and *C*_2_ (mol/L) represent the concentrations of Co[Cyanex 272]_2_ and [C_4_H_9_NH_3_][Cyanex 272] in the organic phase. The *C*_3_ and *C*_4_ (mol/L) represent the concentration of ${\left[C4H9\mathrm{NH}3\right]}_{}^{+}$ and ${\mathrm{Co}}_{}^{+}$ in the aqueous phase. The distribution ratio of the two phases for Co^2+^(*D*_Co_) can be listed as Equation (8).

(8) $$D_{\mathrm{Co}}=\frac{C_{1}}{C_{4}}$$

Equation (9) can be obtained by taking the *D*_Co_ into the *K*_Co_.

(9) $$K_{\mathrm{Co}}=\frac{D_{\mathrm{Co}}\cdot {\left(C_{3}\right)}^{m}}{{\left(C_{2}\right)}^{m}}$$

Equation (10) can be obtained by taking the logarithm of *K*_Co_

(10) $$\mathrm{lg}D_{\mathrm{Co}}=\mathrm{lg}K_{\mathrm{Co}}-m\mathrm{lg}(\frac{C_{3}}{C_{2}})$$

**Figure 6:**
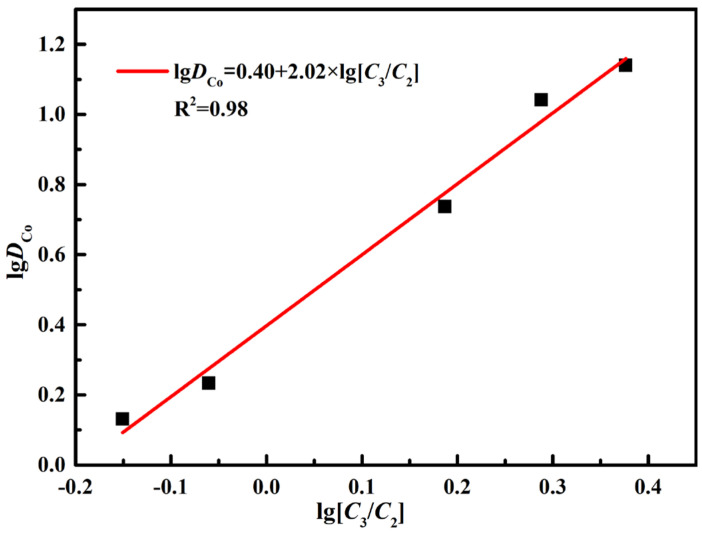
Slope method for the extractive reaction.

The value of *m* in Equation (10) can be determined by the slope method (Figure 6). The *m* is 2.02. The value of *m* is approximately 2.0. Therefore, the equation of chemical reaction can be listed as Equation (11).

(11) $$2[C_{4}H_{9}{\mathrm{NH}}_{3}{][\mathrm{Cyanex}\;272]}_{\left(\mathrm{org}\right)}+{\mathrm{Co}}_{\left(\mathrm{aq}\right)}^{2+}\overset{K_{\mathrm{eq}}}{⇄}{\mathrm{Co}[\mathrm{Cyanex}\;272]}_{2}{}_{\left(\mathrm{org}\right)}+2{\left[C_{4}H_{9}{\mathrm{NH}}_{3}\right]}_{\left(\mathrm{aq}\right)}^{+}$$

### 3.6. Stripping for TSIL-Based Extraction Phase and Its Recycling Use

**Figure 7:**
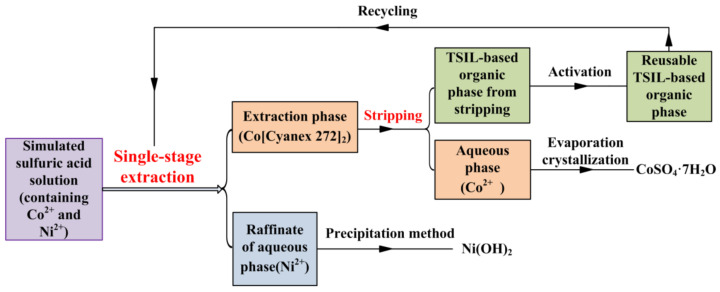
Recycling use of TSIL-based extraction phase.

After single-stage extraction, the TSIL-based extraction phase containing Co[Cyanex 272]_2_ can be stripped with 0.5 mol/L H_2_SO_4_ (Figure 7). The stripping percentage of Co^2+^ from TSIL-based extraction phase was about 99.2% with the optimized stripping conditions, i.e., stripping phase ratio of 1:1, stripping time of 35 min, and stripping temperature of 308 ± 1 K. The product of CoSO_4_·7H_2_O was obtained from the stripping phase using evaporation crystallization [44]. The product of Ni(OH)_2_ can be obtained from the raffinate of the aqueous phase containing Ni^2+^ using the precipitation method [45]. The TSIL-based extraction phase can be recycled for extraction after activation. The specific steps for activation are as follows: (1) the TSIL-based organic phase from stripping is firstly washed using the deionized water (the volume ratio of deionized water to ILs-based organic phase was 1:4), and the aqueous phase containing metal ions, which remained in the organic phase, is completely washed by new deionized water; (2) the reusable TSIL-based organic phase is obtained by the activating reaction of the washed organic phase and commercial n-butylamine solution (the reaction was similar to the Section 2.3), and the recycled TSIL-based organic phase (about 96.4% yield) can be reused for the single-extraction process. 

## 4. Conclusions

In this study, the optimal extraction conditions of Co^2+^ from Ni^2+^ were determined using the RSM method to be: pH_ini_ of 3.7, *t*_e_ of 55.8 min, and a *T*_e_ of 330.4 K. The real yield of *β*_Co/Ni_ obtained by the validation test was 66.1, and the error was 1.7%, which was very close to the predicted value of 67.2. The result shows that the equation is efficient and feasible, with practical significance. The obtained Equation (5) of single-stage extraction is useful to design the multi-stage extraction for efficient separation of Co^2+^ from Ni^2+^_,_ and the multi-stage extraction can be used in industrial applications. The chemical reactive equation was determined using the slope method. Additionally, the recycled TSIL-based organic phase with 96.4% yield can be reused for the single-extraction process.

## Figures and Tables

**Table 1:** Independent variables and levels used for Box–Behnken design.

No.	Variables	Level		
		−1	0	1
*A*	pH_ini_	1.50	3.75	6.00
*B*	*t*_e_ (min)	15.00	52.50	90.00
*C*	*T*_e_(K)	298.15	315.65	333.15

**Table 2:** Box–Behnken design for independent variables and observed responses.

Run No.	pH_ini_	*t*_e_/min	*T*_e_/K	*β*_Co/Ni_
1	3.75	52.50	315.65	64.41
2	1.50	52.50	333.15	54.72
3	3.75	52.50	315.65	63.27
4	3.75	90.00	333.15	48.67
5	3.75	15.00	298.15	29.71
6	3.75	90.00	298.15	40.21
7	3.75	52.50	315.65	65.51
8	6.00	52.50	333.15	55.82
9	1.50	52.50	298.15	42.26
10	6.00	15.00	315.65	27.21
11	6.00	90.00	315.65	38.27
12	1.50	90.00	315.65	34.46
13	1.50	15.00	315.65	22.87
14	3.75	52.50	315.65	64.72
15	6.00	52.50	298.15	44.15
16	3.75	52.50	315.65	63.89
17	3.75	15.00	333.15	36.42

When concentration of [C_4_H_9_NH_3_][Cyanex 272] was set as 0.24 mol/L and O/A = 1:1.

**Table 3:** ANOVA of response quadratic model analysis for the *β*_Co/Ni._.

Scheme	*F*-Value	*p*-Value (Prob > *F*)
Equation (5)	159.5146	<0.0001 ^a^
*A*	6.691242	0.0361
*B*	111.1343	<0.0001
*C*	83.27631	<0.0001
*AB*	0.030291	0.8668
*AC*	0.067301	0.8028
*BC*	0.33025	0.5835
*A* ^2^	243.8083	<0.0001
*B* ^2^	884.7419	<0.0001
*C* ^2^	22.71165	0.0020

*F*-value is from the *F*-test, which is most commonly known as the Joint Hypotheses test, and it is a test under the Null hypothesis; *p*-value is a hypothesis probability and an important basis for judging the significance of factors in statistics; ^a^: significant.

**Table 4:** Comparison of separation factor of Co^2+^ from Ni^2+^ using single-stage extraction.

Extractants	Diluent	Main Conclusion
Cyphos IL 104 [4]	Exxsol D80 or toluene	*β*_Co/Ni_ is about 14 with optimized extraction conditions, i.e., 0.2M Cyphos IL 104, O/A = 1:1, *t*_e_ of 15 min, and *T*_e_ of 296 ± 2 K
Cyanex 301 [39]	Kerosene	*β*_Co/Ni_ is about 1.8 with optimized extraction conditions, i.e., 1.5M Cyanex 301, O/A = 1:1, pH_ini_ of 1, *t*_e_ of 30 min, and *T*_e_ of 298 ± 1 K
Primene JMT-Cyanex 272 [13]	Exxol D100	*β*_Co/Ni_ is about 45.3 with optimized extraction conditions, i.e., 1:1 percentage composition of JMT-Cy272, 0.4M chloride solution, *t*_e_ of 20 min, and *T*_e_ of 296 ± 3 K
[C_4_H_9_NH_3_][Cyanex 272] (Paperwork)	n-Hexane	*β*_Co/Ni_ is about 67.2 with optimized extraction conditions, i.e., 0.24M [C_4_H_9_NH_3_][Cyanex 272], O/A = 1:1, pH_ini_ of 3.7, *t*_e_ of 55.8 min, and *T*_e_ of 330.4 K,

## Supplementary Materials

The following supporting information can be downloaded at: https://www.mdpi.com/article/10.3390/molecules27154806/s1, Figure S1: title: ^1^H NMR of C_4_H_9_NH_2_; Figure S2: title: ^1^H NMR of [C_4_H_9_NH_3_][Cyanex 272]; Figure S3: title: ^13^C NMR of [C_4_H_9_NH_3_][Cyanex 272]; Figure S4: title: FT-IR of [C_4_H_9_NH_3_][Cyanex 272]; Figure S5: title: Homogenous phase of mixing of C_4_H_9_NH_2_ and Water; Figure S6: title: Two insoluble phases of mixing of [C_4_H_9_NH_3_][Cyanex 272] and Water.

## Sample Availability

Not available.
