# Supplementary material for: Single-Stage Extraction and Separation of Co2+ from Ni2+ Using Ionic Liquid of [C4H9NH3][Cyanex 272]
Source: Molecules. 2022 Jul 27;27(15):4806. doi: 10.3390/molecules27154806 (PMC9369997; doi:10.3390/molecules27154806)
Supplement: Supplementary file 1 [file molecules-27-04806-s001.zip › molecules-1816669-supplementary.pdf]

Supplementary materials

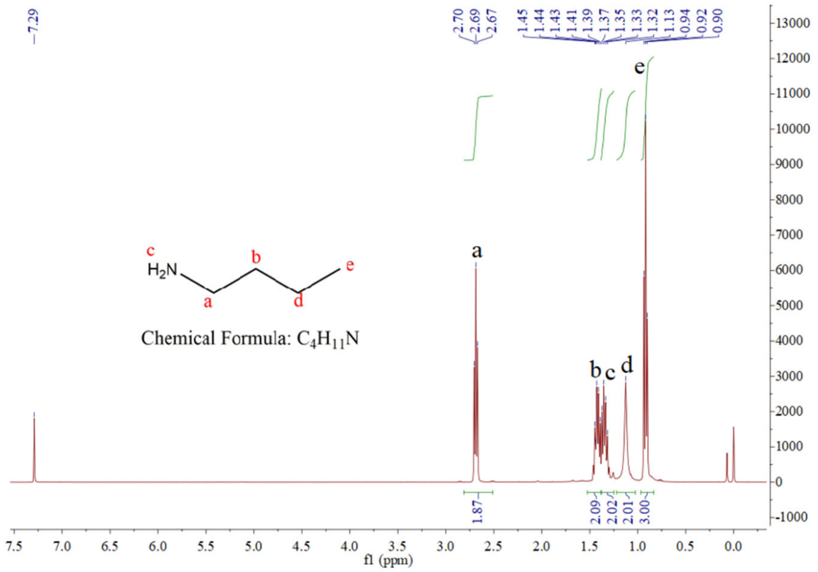

Figure S1. ^{1}H NMR of $C_4H_9NH_2$.

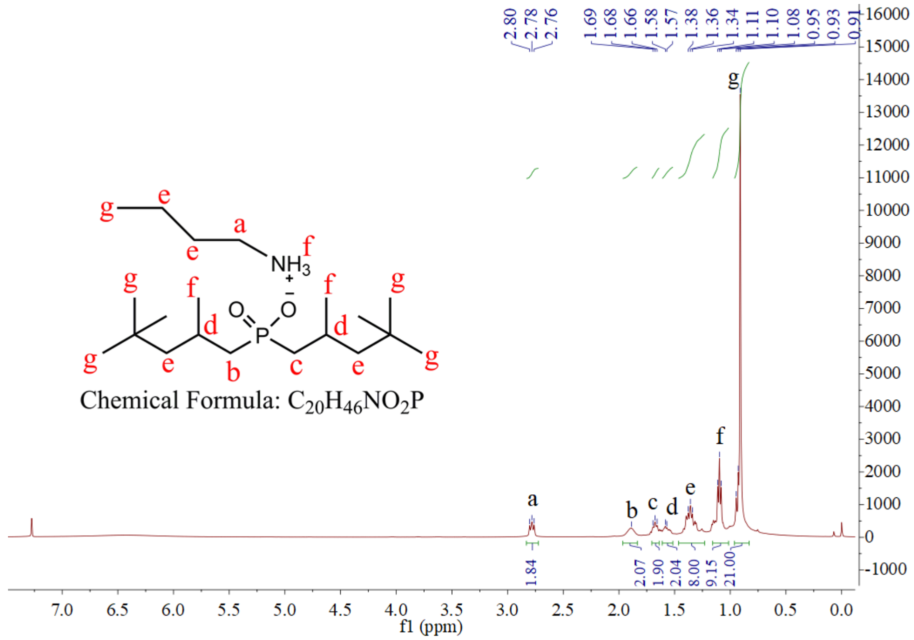

Figure S2. ^{1}H NMR of $[C_4H_9NH_3]$[Cyanex 272].

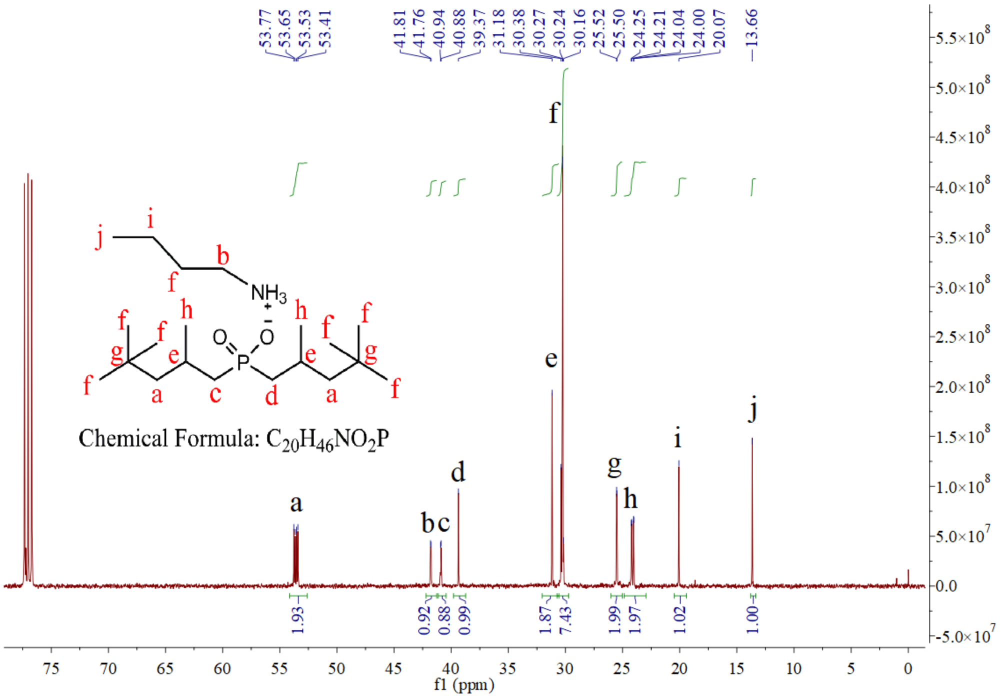

Figure S3. ^{13}C NMR of $[C_4H_9NH_3]$[Cyanex 272].

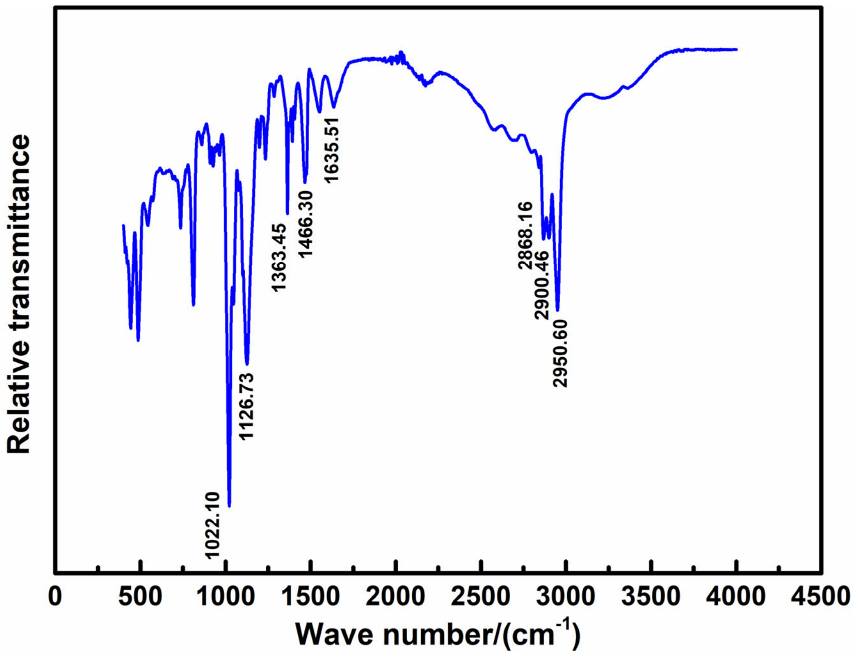

Figure S4. FT-IR of $[C_4H_9NH_3]$[Cyanex 272].

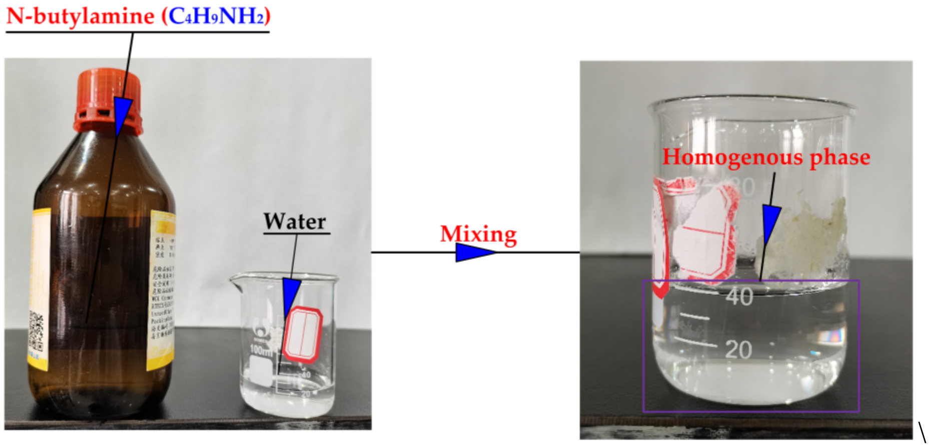

Figure S5. Homogenous phase of mixing of $C_4H_9NH_2$ and Water.

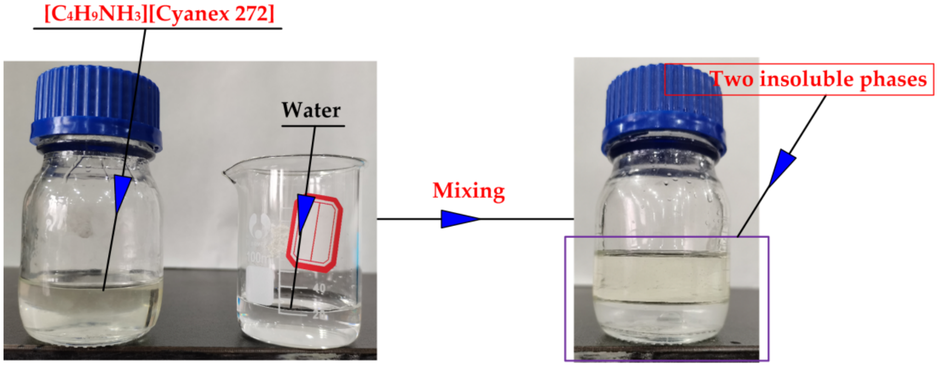

Figure S6. Two insoluble phases of mixing of $[C_4H_9NH_3]$[Cyanex 272] and Water.
